# MetaRNA-Seq: An Interactive Tool to Browse and Annotate Metadata from RNA-Seq Studies

**DOI:** 10.1155/2015/318064

**Published:** 2015-08-25

**Authors:** Pankaj Kumar, Anna Halama, Shahina Hayat, Anja M. Billing, Manish Gupta, Noha A. Yousri, Gregory M. Smith, Karsten Suhre

**Affiliations:** ^1^Weill Cornell Medical College in Qatar, Education City, Doha, Qatar; ^2^Institute of Bioinformatics and System Biology, Helmholtz Zentrum Munchen, Germany Research Center of Environmental Health, 85764 Neuherberg, Germany

## Abstract

The number of RNA-Seq studies has grown in recent years. The design of RNA-Seq studies varies from very simple (e.g., two-condition case-control) to very complicated (e.g., time series involving multiple samples at each time point with separate drug treatments). Most of these publically available RNA-Seq studies are deposited in NCBI databases, but their metadata are scattered throughout four different databases: Sequence Read Archive (SRA), Biosample, Bioprojects, and Gene Expression Omnibus (GEO). Although the NCBI web interface is able to provide all of the metadata information, it often requires significant effort to retrieve study- or project-level information by traversing through multiple hyperlinks and going to another page. Moreover, project- and study-level metadata lack manual or automatic curation by categories, such as disease type, time series, case-control, or replicate type, which are vital to comprehending any RNA-Seq study. Here we describe “MetaRNA-Seq,” a new tool for interactively browsing, searching, and annotating RNA-Seq metadata with the capability of semiautomatic curation at the study level.

## 1. Introduction

High-throughput gene expression studies are pivotal in functional biology and genomics research [1]. Until late in the last decade, high-throughput gene expression studies were carried out using microarray technology [2], which has provided great insight into several gene expression studies and led to the establishment of public repositories, such as the Gene Expression Omnibus (GEO) hosted by the NCBI [3, 4]. With the advent of next-generation sequencing (NGS) technology and its meteoric growth [5, 6], RNA-Seq is slowly becoming prevalent in high-throughput gene expression studies [7]. RNA-Seq studies have several advantages over microarray technology, including whole transcriptome analysis, better reproducibility, and a larger dynamic range of expression [8, 9]. Initially, RNA-Seq studies were deposited in the GEO database. However, because the type and amount of data are similar to other NGS data, RNA-Seq studies are now deposited in the Sequence Read Archive (SRA) [10]. The metadata from projects in RNA-Seq studies are hosted by the NCBI Bioproject database. The metadata for samples used in RNA-Seq studies are deposited in the NCBI Biosample database. The metadata often lack sufficient information because the submitters limit themselves by providing only the mandatory information. In addition, some of the important metadata for these studies are not deposited into these repositories but are found in the publications resulting from these studies. Thus, biocuration of RNA-Seq metadata is needed.

The importance of biocurating biological databases was realized in recent years [11, 12]. Text mining and computer-assisted biocuration of the literature has helped to create curated biological databases [13–17]. In the case of RNA-Seq metadata annotation, consensus summaries of underlying biosamples, experiments, and runs at the study level are helpful. Currently, most of the submitter-provided field attributes or annotation information in NCBI RNA-Seq repositories is linked to individual biosamples, experiments, or runs. The important attributes describing the overall study, such as disease type, study type, and replicate type, are not available through the NCBI interface or repositories. A clear description of study types, such as case-control and time series, would allow users to easily comprehend a RNA-Seq study. Similarly, the metadata for a RNA-Seq study should clearly note whether a study was done to dissect a disease (e.g., study designed to find differentially expressed genes in diseased versus normal individuals), which is often uncertain because of the biosample types used in the study. The default search interface of the NCBI SRA database provides experiment-level results and the possibility of going to biosample, study, run, or bioproject pages. Users searching for a particular type of RNA-Seq study in these repositories may have to invest a lot of time because of non-annotated fields and go back and forth through multiple hyperlinks to obtain the desired information. It is critical for researchers to have an alternative method of evaluating RNA-Seq metadata supplemented with manual curation. Here we introduce a new tool called “MetaRNA-Seq” to interactively browse, search, and annotate RNA-Seq metadata at the study level. MetaRNA-Seq provides an easy to use web interface to understand metadata for RNA-Seq studies. Most of the details about any RNA-Seq study are provided in the same window with a single click. MetaRNA-Seq provides a consensus summary for any RNA-Seq study by digesting all biosample, experiment, and run information for that study. In addition, MetaRNA-Seq provides the hierarchical data of the study in a tree-like structure. In MetaRNA-Seq, the metadata for a RNA-Seq study can be annotated and searched based on annotated fields, such as disease type, time series, case-control, replicate type, and customized annotation. MetaRNA-Seq is available at http://metarnaseqdb.screensifter.com/.

## 2. Materials and Methods

The metadata for RNA-Seq studies from multiple resources at NCBI were retrieved using the NCBI entrez direct utility [18]. Scripts were written to query the NCBI SRA database, which utilizes the “esearch” tool of the NCBI entrez direct utility with the search term “biomol rna [PROP] AND Homo sapiens [orgn:___txid9606] AND (RNA-seq or rnaseq).” Next, the “efetch” and “xtract” tools of the NCBI entrez direct utility were used to fetch the metadata available from the SRA database and to parse them. Attributes retrieved from these parsed result sets, such as Biosample Ids and Bioproject Ids, were used to query the NCBI Biosample and Bioproject databases using the NCBI entrez direct utility. These data were imported into the MySQL database and indexed for quick access. Additional metadata for RNA-Seq studies with external database records in GEO were retrieved using GEOmetadb [19]. The web technology used in MetaRNA-Seq is the Java EE 7 and VAADIN framework. Glassfish is used as a webserver to host the MetaRNA-Seq web application. The suggested automatic biocuration provided in MetaRNA-Seq is simple text mining. Compared to Natural Language Processing- (NLP-) based advanced biocuration algorithms adopted in tools like MyMiner and Pubtator [14, 17], MetaRNA-Seq utilizes simple regular expression. Suggestions for RNA-Seq study-level annotation are based on the regular expression-based match statistics, such as number of biosamples, experiments, or runs with matching keywords out of the total number of biosamples, experiments, or runs. The MetaRNA-Seq database is updated from the NCBI RNA-Seq databases using a similar method as when initially fetching the data through the NCBI entrez direct utility and new records are inserted into MySQL tables.

## 3. Results

**Figure 1:**
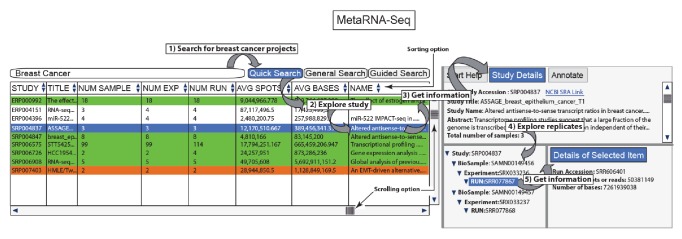
The MetaRNA-Seq web interface. On the left it has the search functionality for RNA-Seq studies. Below the search, the table contains all RNA-Seq study details, including name, title, number of samples, number of experiments, and number of runs, allowing one to quickly scroll through all of the studies. The table is filtered based on the search. The table can be sorted by double clicking any column. Upon clicking any study in the table, the study details are populated at the upper right. A tree-like data structure containing biosamples, experiments, and runs for the selected study is populated in the lower right.

The MetaRNA-Seq web tool provides enhanced utilization of metadata for RNA-Seq studies in NCBI resources with semiautomatic curation, restructures the presentation, and allows convenient browsing of all of the details in a single window with a study-level search. MetaRNA-Seq currently has metadata information for 1508 human RNA-Seq studies and is updated every quarter. The main web interface of MetaRNA-Seq is shown in Figure 1. The interface has a desktop application-like design and behavior.

### 3.1. Study-Level Presentation of RNA-Seq Studies

In contrast to the NCBI SRA interface, which by default provides an experiment-level view of the results of a search query, MetaRNA-Seq presents a study-level browsing and filtering mechanism. All RNA-Seq studies are presented in a table in the MetaRNA-Seq web interface. The table contains important columns, such as study accession, name, title, number of samples, number of experiments, number of runs, and average number of reads and bases. The table is filtered based on the search. The studies table can be sorted by double clicking any column. Study details appear for any selected project, including title, name, abstract, material, method, bioproject description, GEO design, and summary. This table of RNA-Seq studies also provides manual annotation details for the RNA-Seq study if that study is annotated using the MetaRNA-Seq annotation interface. In addition, the MetaRNA-Seq interface provides hyperlinks to NCBI SRA or PubMed if the user needs additional information about a study.

### 3.2. Annotation Capability

**Figure 2:**
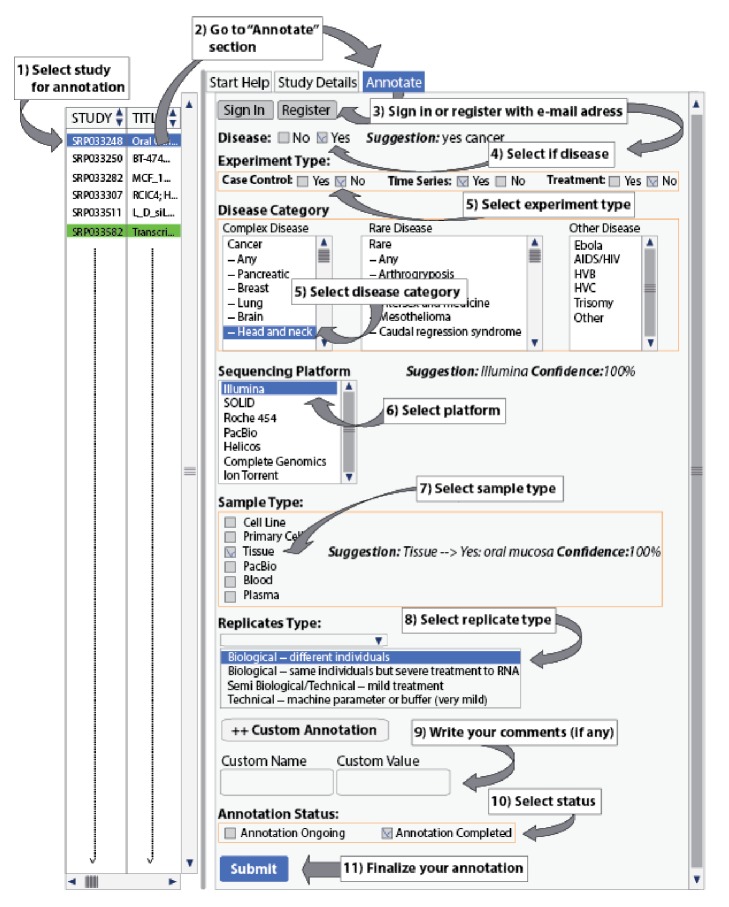
RNA-Seq metadata annotations in MetaRNA-Seq. Suggestions are based on a program-assisted search of all data available for a particular study. Custom annotation fields can be used in cases when the annotator feels that additional information is important and it cannot be stored using default options. The annotator can use as many custom annotation fields as required.

MetaRNA-Seq has a semiautomatic curation capability and provides an easy interface for annotating metadata for RNA-Seq studies, mostly with one mouse click (Figure 2). Categorization of most of the annotation fields is based on their impact and effect on RNA-Seq studies [20–23]. One can intuitively determine that transcript expression profiles in a case-control study involving a rare disease will be very different than a case-control study involving a complex disease. In MetaRNA-Seq, a RNA-Seq study can be annotated for study type, disease category, sample type, replicate type, and custom annotation. Study types are categorized broadly into case-control, time series, and treatment. Sample types are categorized into cell line, primary cells, tissue, blood, and plasma. If some of these fields are present in experiments or biosamples in the selected study, then the assistance is automatically generated with a certain degree of confidence. For example, while annotating a RNA-Seq study with accession “SRP010129,” automatic suggestions of cell line and tissue are provided for the sample types with hint text “***Suggestion:*** Cell Lines - -> Yes: Cell line derived from Merkel Cell Carcinoma (10 samples) ***Confidence:*** *25%*” and hint text “***Suggestion:*** Tissue - -> Yes: FFPE Merkel Cell Carcinoma (16 samples), FFPE Basal Cell Carcinoma (6), FFPE Normal skin (6), FFPE Squamous Cell Carcinoma (2) ***Confidence:*** *40%*,” respectively (Figure 2). The automatic hint provision can help annotators perform annotation more quickly in MetaRNA-Seq. Also, study type, sample type, or platform can be selected for multiple types. For example, in a complex RNA-Seq study, the study type can be both case-control and time series, sample types can be both cell line and primary cells, and platforms can be Illumina, Solid, and Roche 454.

### 3.3. Guided Search

**Figure 3:**
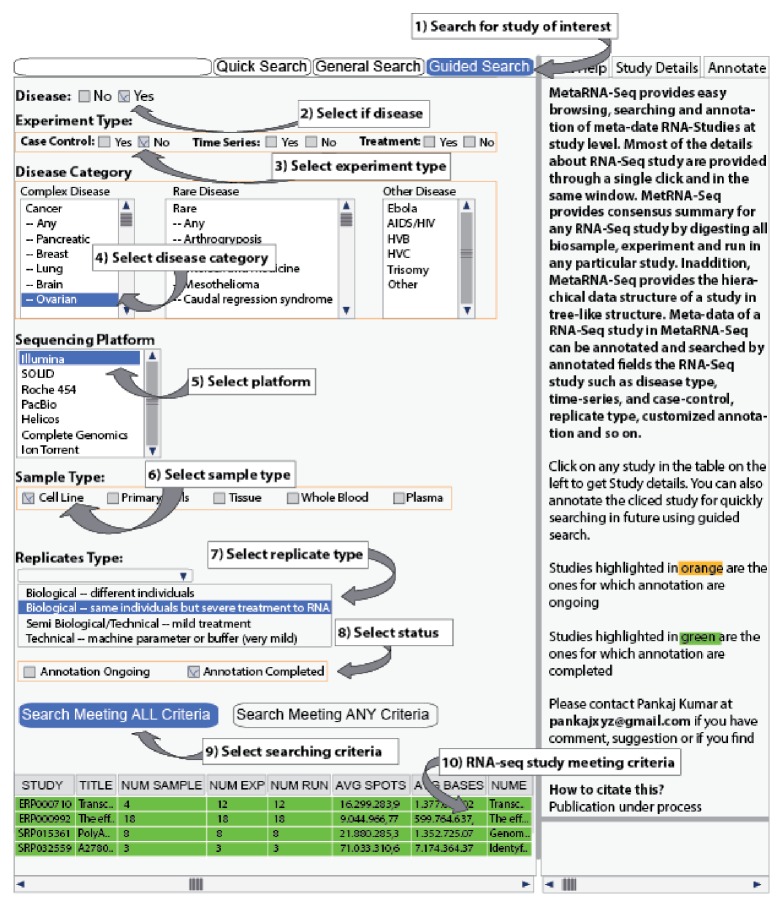
Guided search using annotated fields. The search is performed to identify RNA-Seq studies involving breast cancer with cell line as the sample type and annotation status as completed. The result output is a filtered table, with rows highlighted in green because it searched for completed annotation. The user can obtain additional details about any of the filtered studies by simply clicking on them.

RNA-Seq studies can be searched precisely for annotated fields using guided search. This can help identify RNA-Seq studies just by clicking various checkboxes and options rather than typing queries into different fields. A search combining multiple fields can be performed easily for studies meeting all or any criteria. Studies in the MetaRNA-Seq study table are filtered based on the search output. One example of searching annotated RNA-Seq studies related to breast cancer with “cell line” as the sample type is shown in Figure 3.

## 4. Discussion

The information from publically available RNA-Seq studies can be exploited to (1) improve experimental settings by comparing different platforms under similar experimental conditions, (2) compare whole transcripts of different cell lines, tissues, diseases, and experimental conditions (e.g., drug treatment and time of drug exposure) to select the most suitable conditions for newly designed experiments, and (3) estimate the number of samples and number of biological and technical replicates needed to obtain significant and relevant data. However, NCBI database interfaces are unable to provide the study-level search and comparisons required by the end user. Using annotated studies with the guided search function provided by MetaRNA-Seq is strongly recommended to supplement NCBI RNA-Seq metadata resources. The annotation capabilities provided in MetaRNA-Seq can be utilized by end users to support the community and improve the search process in subsequent sessions. MetaRNA-Seq can easily be turned into a crowd sourcing-based annotated resource. Currently, the interface provides all of the annotation details for the studies with annotation completed by all biocurators, as the number of biocurators is limited to our lab at the time of writing this paper. In the future, if the number of biocurators increases or if multiple biocurators handle a single study, manual annotation study details can be presented based on crowd statistics. MetaRNA-Seq currently presents the metadata for RNA-Seq studies of* Homo sapiens* as a prototype, but it can be expanded to include RNA-Seq metadata from other species in subsequent versions. Tools like MetaRNA-Seq are necessary to supplement big repositories, which make it difficult to focus on a specialized topic and may even provide them with ideas on implementation and execution, which may stimulate the big repositories to implement features such as a simple, click-based annotation interface.

## 5. Conclusion

The NCBI provides many options for finding RNA-Seq data. However, the large amount and complex nature of RNA-Seq data that can be retrieved using NCBI data resources present difficulties for researchers. An annotated resource of RNA-Seq metadata is needed to better serve the community. A tool such as MetaRNA-Seq can be a great resource for both annotating and browsing RNA-Seq metadata, and it can provide better tools for more effective data interrogation. Enhanced access to RNA-Seq metadata could also potentially allow the creation of a customized RNA-Seq metadata database and has the potential to be turned into a crowd sourcing-based annotated resource for the benefit of the research community.
